# Supplementary material for: Integrative analyses of the RNA modification machinery reveal tissue- and cancer-specific signatures
Source: Genome Biol. 2020 May 7;21:97. doi: 10.1186/s13059-020-02009-z (PMC7204298; doi:10.1186/s13059-020-02009-z)

Additional File 2: Begik et al, 2020

Fig. S1. (A) Heatmap of z-scaled log2 TPM values of RMPs from human and mouse tissues. RMPs have been subdivided into 7 different classes depending on their annotated function: i) methylases, ii) deaminases, iii) pseudouridylases, iv) other writer activity, v) non-catalytic subunit, vi) readers and vii) erasers. RMPs have been individually clustered within each class. **(B)** Principal Component Analysis (PCA) of human and mouse tissues based on the expression of their RMPs across tissues. Only the first two principal components are shown. Variance explained by each principal component are shown in each axis. In the loadings plots, RMPs are colored following the same classification used in panel A.

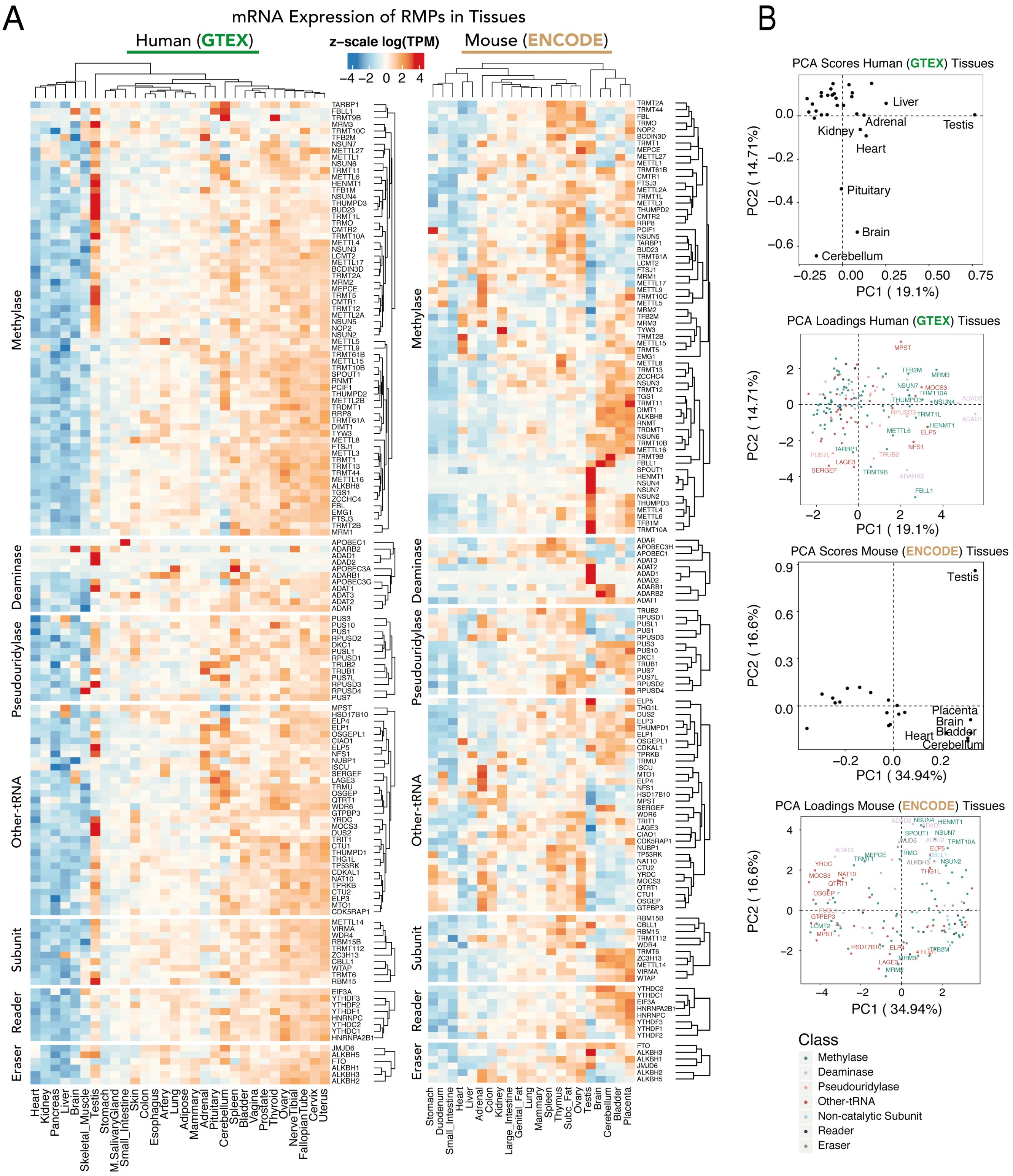

Fig. S2. Quantitative real-time PCR of 8 RMPs expressed in four mouse tissues (brain, liver, lung and testis) normalized to either GAPDH **(A)** or METTL5 **(B)**. RMPs have been grouped into two categories, based on whether they are tissue-specific/enriched or non-tissue specific/enriched, as per RNAseq analysis. All the tissues are normalized to the brain tissue of their corresponding biological replicate.

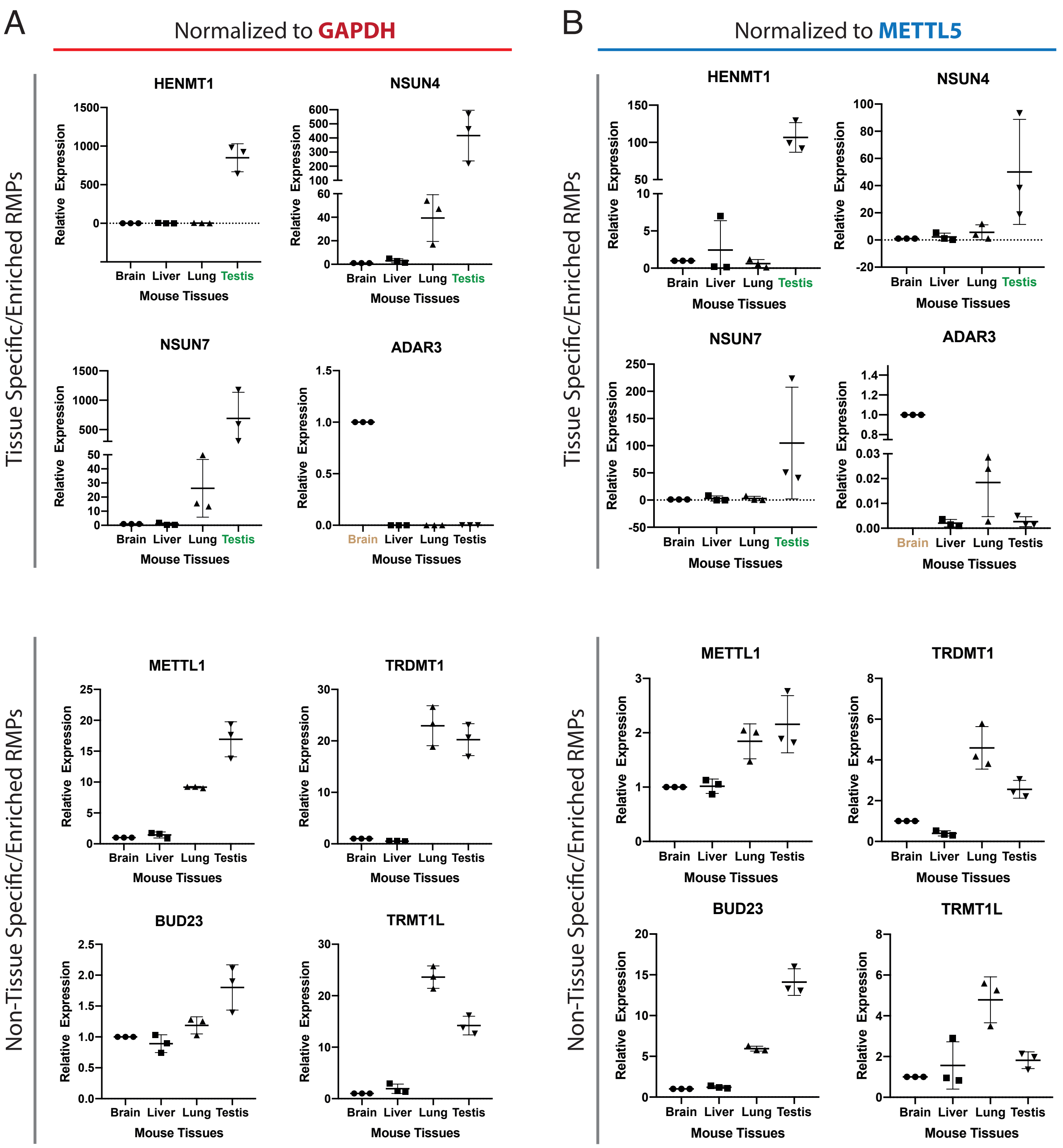

Fig. S3. (A) Heatmap of z-scaled log2 protein levels of RMPs in human tissues. RMPs have been subdivided into 7 classes depending on their annotated function: i) methylases, ii) deaminases, iii) pseudouridylases, iv) other writer activity, v) non-catalytic subunit, vi) readers and vii) erasers. RMPs have been individually clustered within each class. **(B)** Scatter plots depicting tissue-specificity analysis based on protein levels, which have been computed by representing the RMP mRNA expression values in a given tissue (y axis) relative to the mean mRNA abundance in all tissues (x axis). Scatter plots show that testis has a significant number of tissue-specific genes in human, whereas colon shows only one tissue-specific gene in human. Tissue-specific genes are labeled in red.

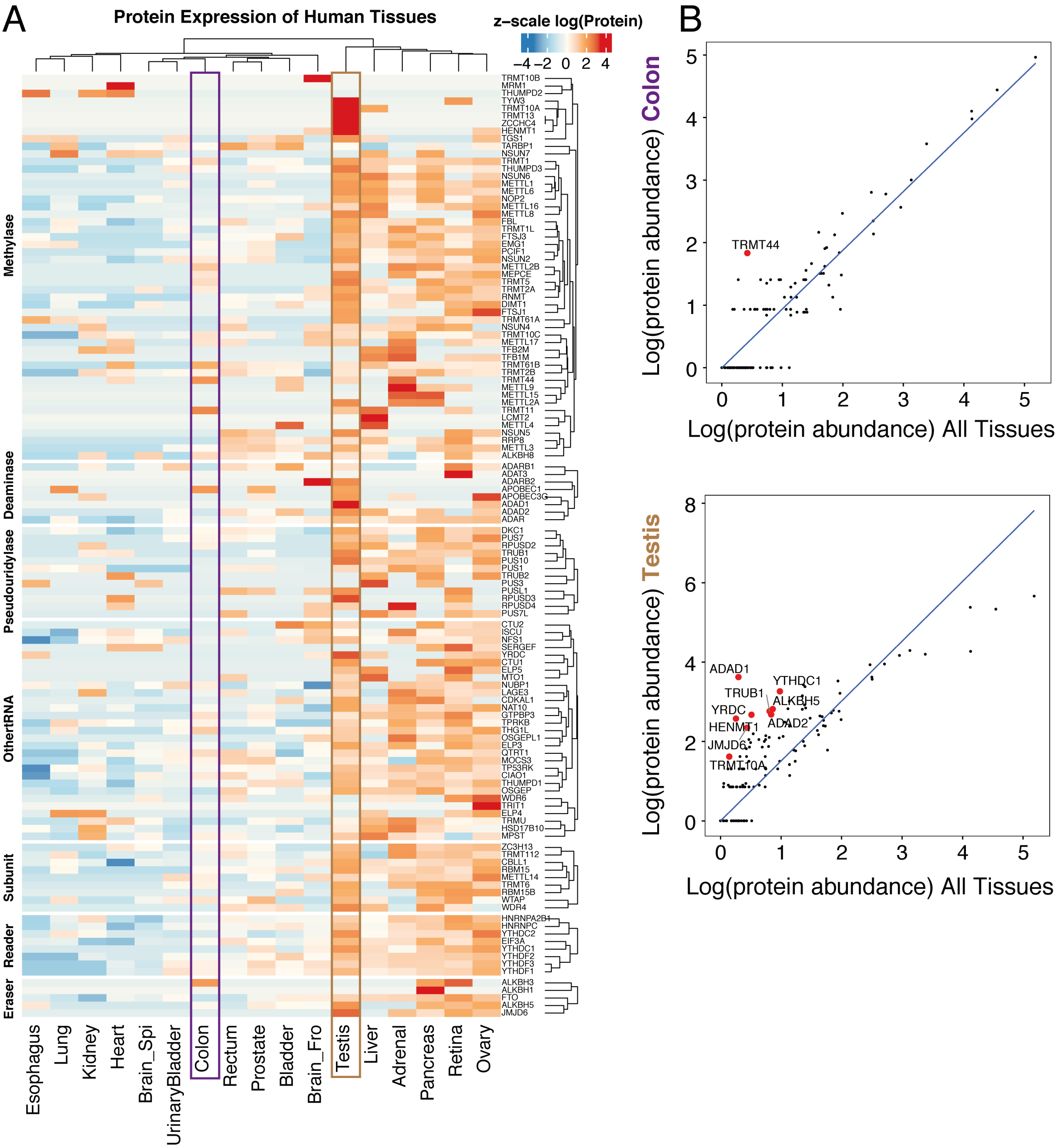

Fig. S4. (A) Barplots depicting HENMT1 and ADAD1 expression values in different amniote species and tissues, showing conserved testis-specific expression of these enzymes. **(B, C)** Principal Component Analysis (PCA) of RMP expression values in primates, using as input the log(RPKM) expression of RMPs. Both scores (B) and loadings (C) are shown for the first two principal components. Variance explained by each PC are shown in each axis**. (D)** Barplots depicting ADARB1 and SERGEF expression values in different primate species and tissues, showing conserved brain- and cerebellum-specific expression of these enzymes.

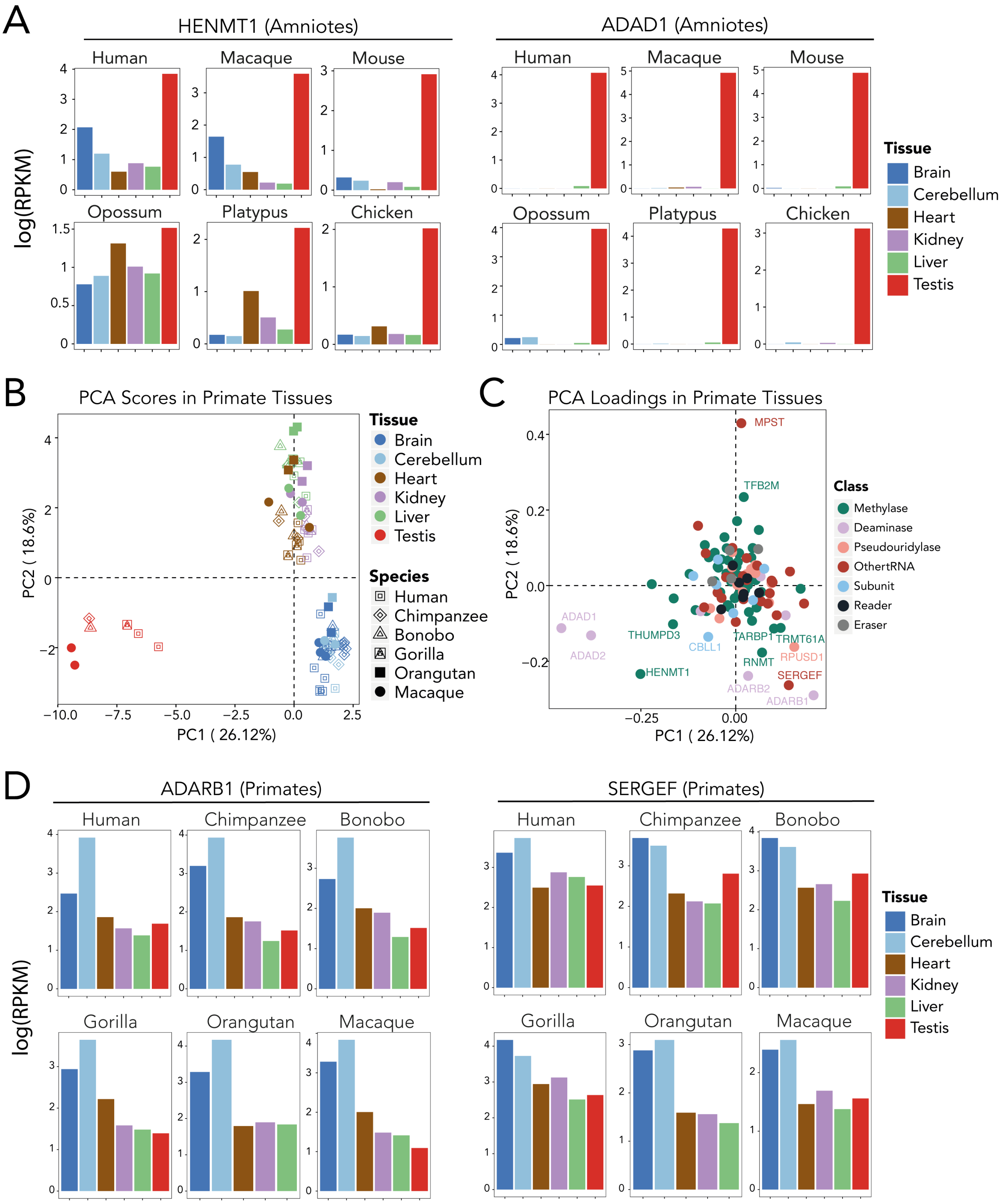

Fig. S5. Analysis of target specificity of tissue specific and non-tissue specific genes. **(A)** Non-tissue specific RMPs mainly target tRNAs, rRNAs and mRNAs. Tissue specific RMPs also target these RNAs, however the proportion of tRNA and rRNA targets is lower. Instead, tissue-specific RMPs target a higher proportion of small non-coding RNAs, including piRNA and eRNA. **(B)** RNA target specificity of tissue-specific RMPs. Only tissues that had 5 or more tissue-specific RMPs were included in the analysis (4/32). The final set of tissues that met this criterion were: testis (n=20), liver (n=6), bone marrow (n=6) and cerebellum (n=8). Liver and bone marrow mainly target tRNAs, rRNAs and mRNAs. In contrast, cerebellum and testis display a larger proportion of tissue-specific RMPs that target distinct families of small non-coding RNAs.

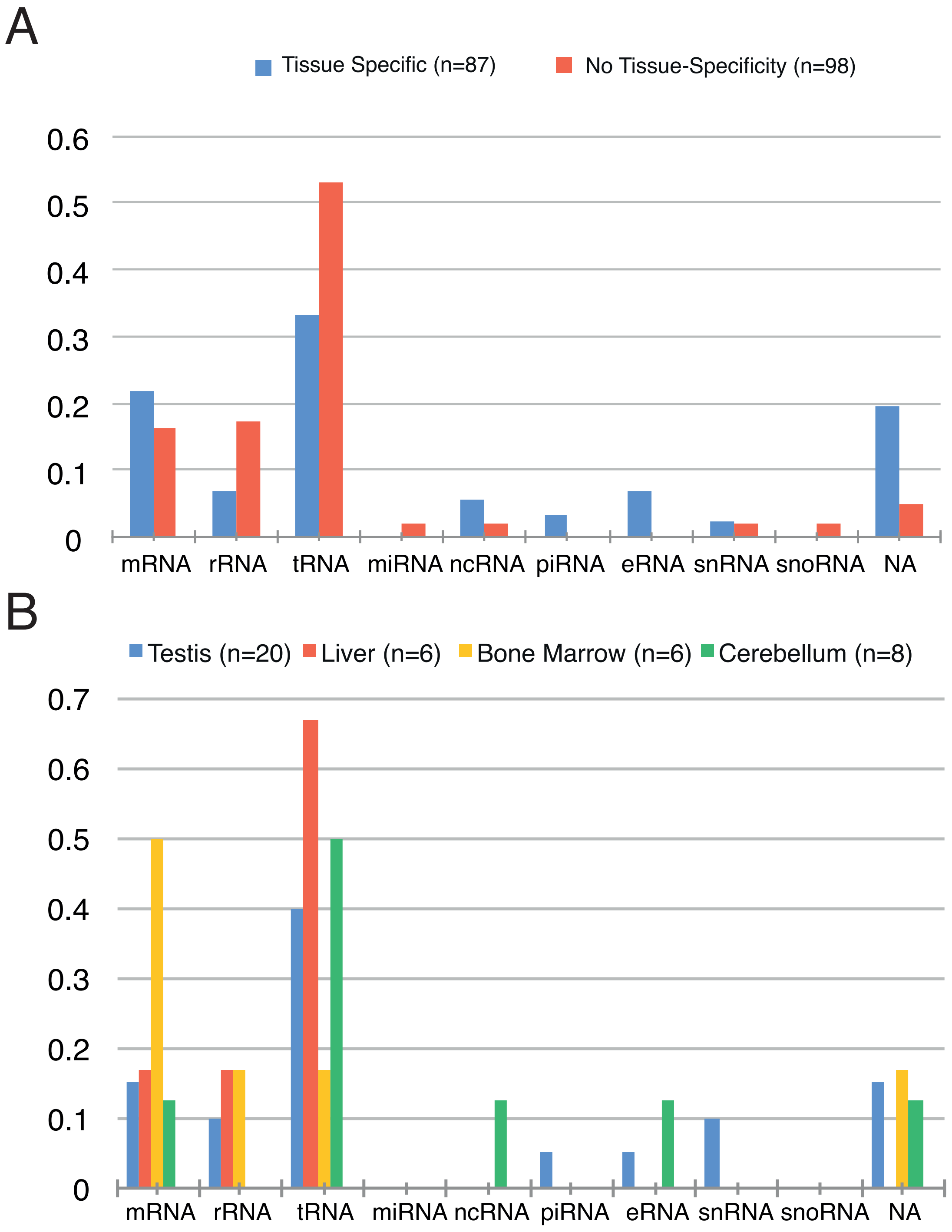

Fig. S6. (A) Heatmap of RMP expression patterns during spermatogenesis, for each of the 4 clusters identified using k-means. **(B)** Within groups, sum of squares was used to determine the optimal number of clusters in spermatogenesis RMP analysis, which is referred to as 'Scree's test'. Based on this test, the optimal number of clusters is 4, which corresponds to the elbow in the curve. **(C)** Principal Component Analysis (PCA) of spermatogenesis RMP expression values. Genes have been colored according to their corresponding cluster. **(D)** Scatter plot depicting tissue-specificity analysis of epididymis tissue, using as input the HPA dataset. Tissue-specific genes are labeled in red. **(E)** In the upper plot, mRNA expression values of HENMT1 for each spermatogenesis maturation stage are shown. In the lower plot, immunostaining of mouse testis and epididymis using HENMT1 antibody is shown. Brown color indicates a specific staining whereas blue shows hematoxylin counterstain. Arrows show subcellular localization of HENMT1.

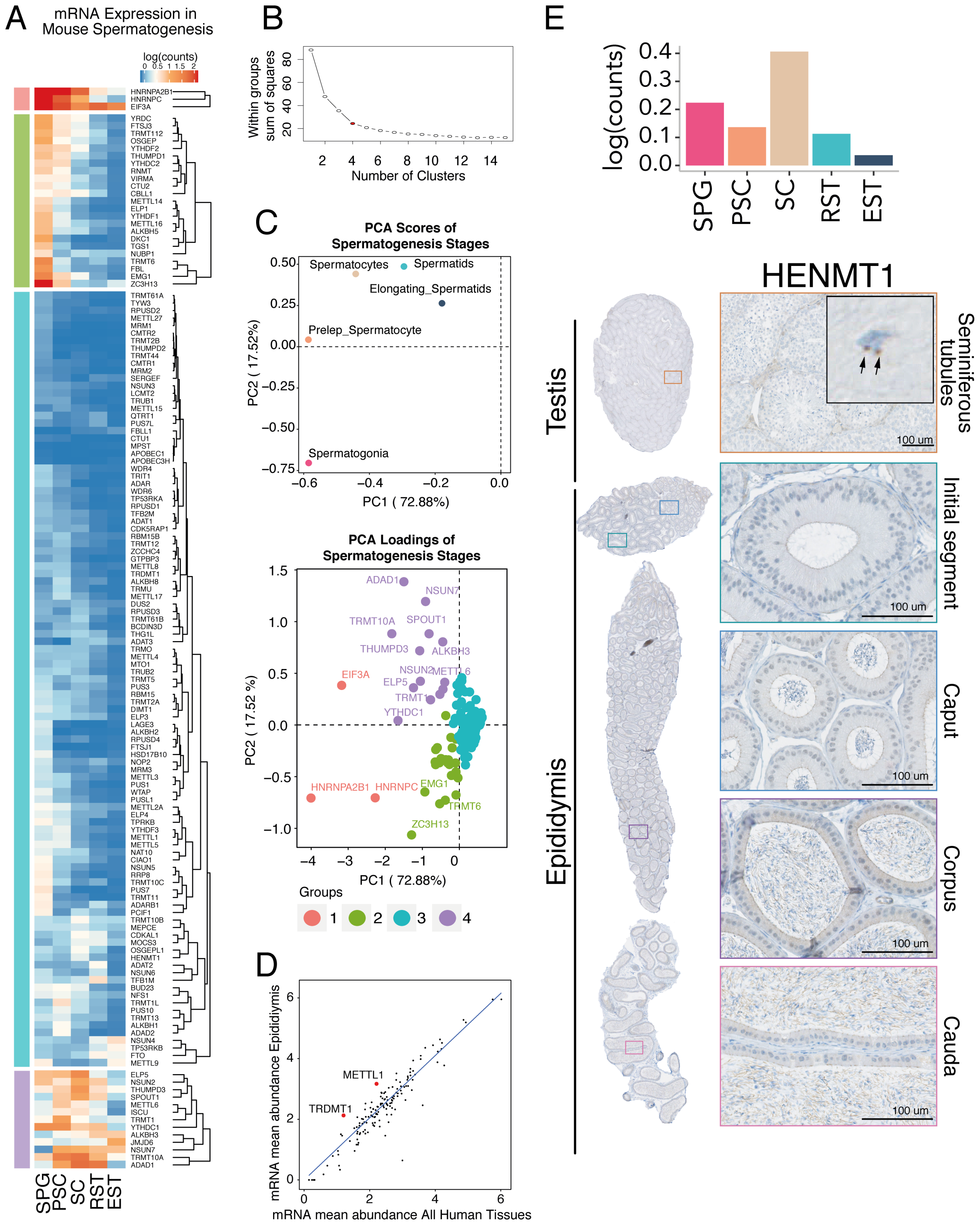

Fig. S7. Comparison of RMP expression changes during spermatogenesis using published single-cell RNA sequencing datasets. Heatmaps and violin plots of log counts shows the expression patterns of different gene clusters based on their expressional behavior during spermatogenesis from **(A)** Green et al., 2018 and **(B)** Xia et al., 2020.

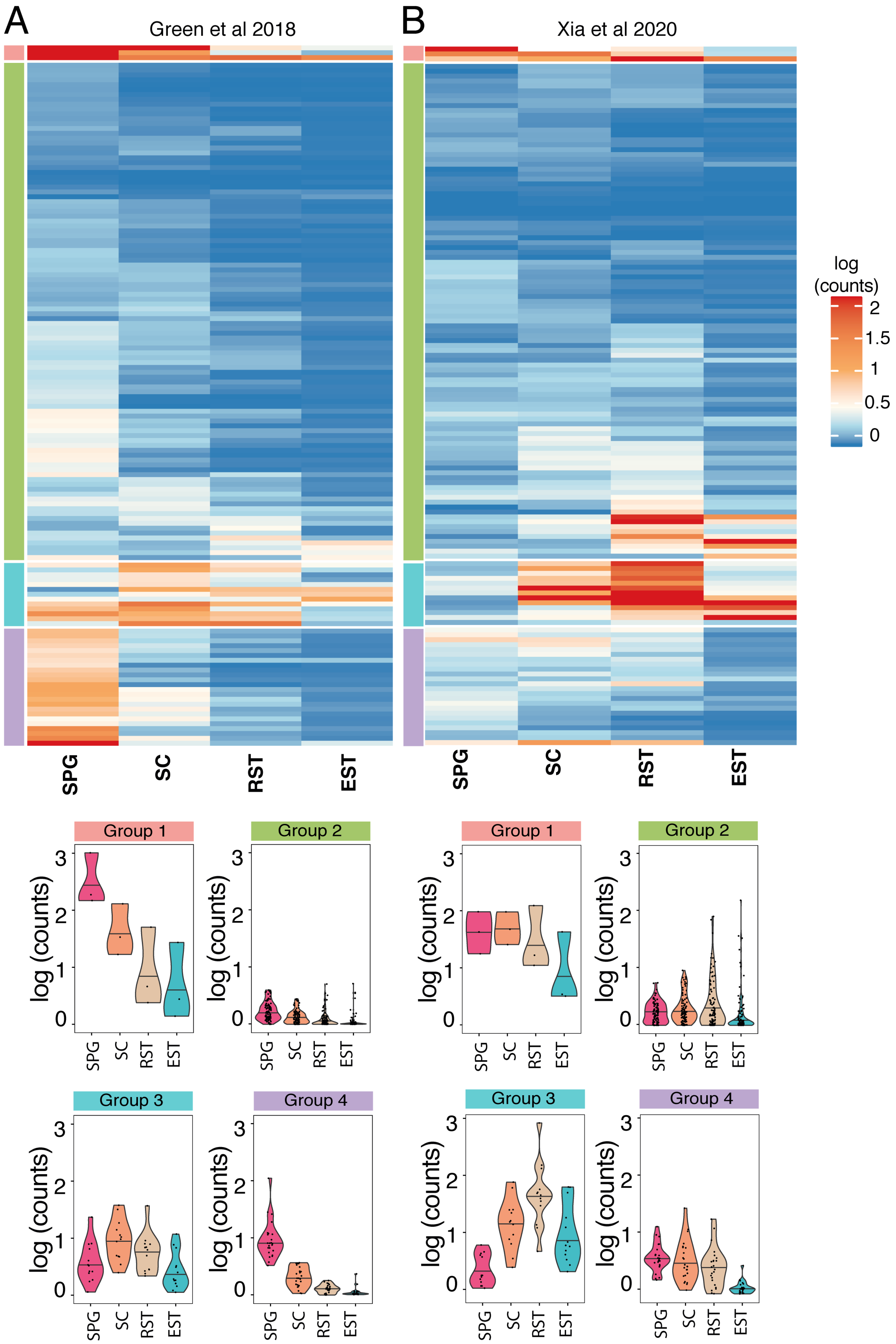

Fig. S8. Comparison of RMP expression patterns during spermatogenesis, using the data published by Green et al., 2018 and Jung & Wells et al., 2019. Gene expression profiles from randomly selected genes – three genes per group identified using the initial analysis of single-cell RNA sequencing data (Green et al., 2018) – were extracted using the interactive website from Jung and Wells et al. 2019.

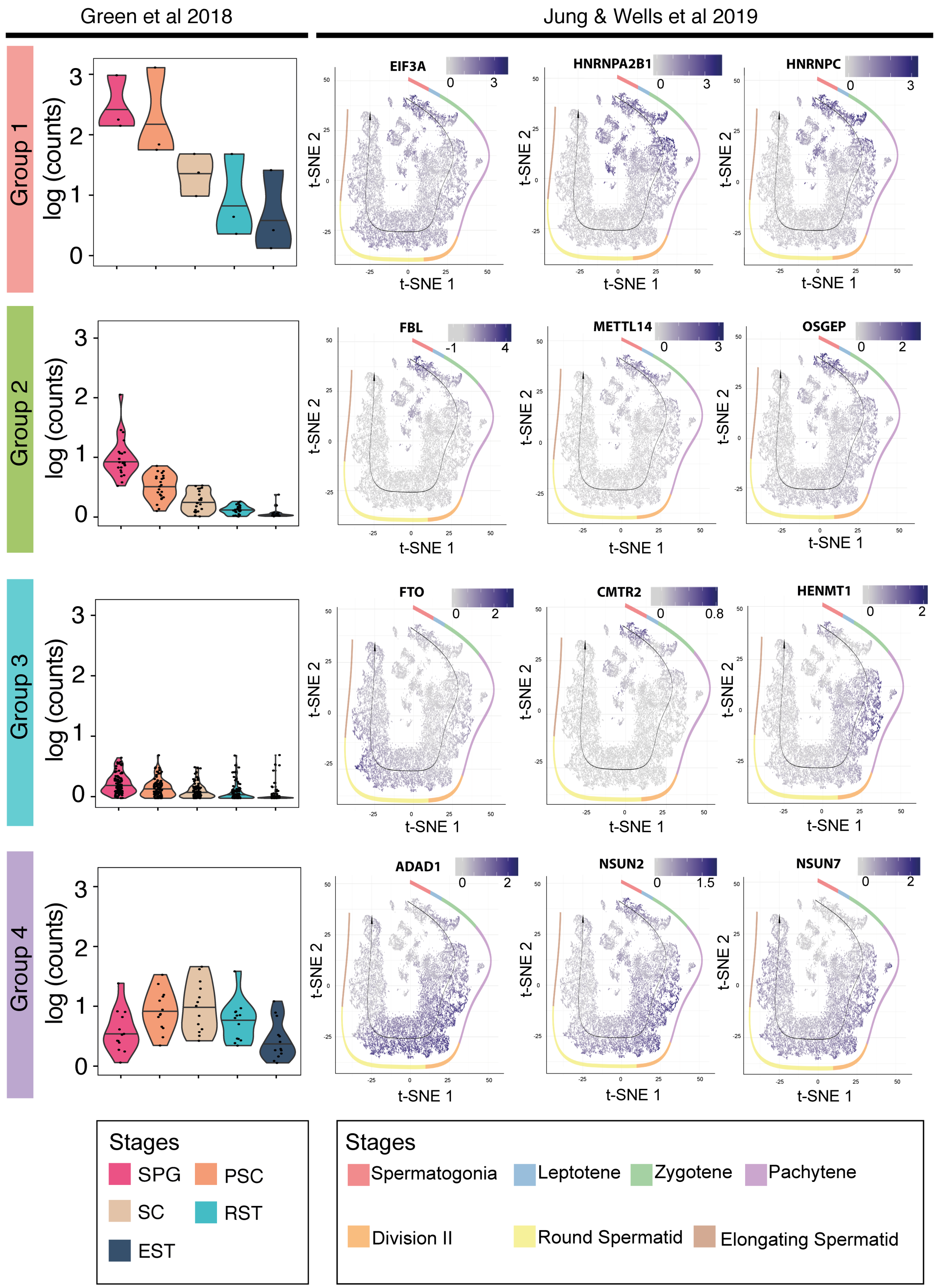

Fig. S9. Comparative analysis of mRNA expression levels of HENMT1, NSUN2, NSUN7 and METTL14 during spermatogenesis, extracted from 3 distinct single-cell RNAseq publicly available datasets. Barplots of log counts shows the expression patterns of different genes during spermatogenesis. Multiple bars in the same group shows different stages within that group along the spermatogenesis at increasing time-points. Expression levels of each of the clusters defined by each work are shown below, and these have been colored according to the following scheme: i) SPG containing spermatogonial cells (pink); ii) SC, contains early and late spermatocyte cells (orange); iii) STD, containing round spermatids (cyan); iv) EST containing elongating spermatids (dark blue).

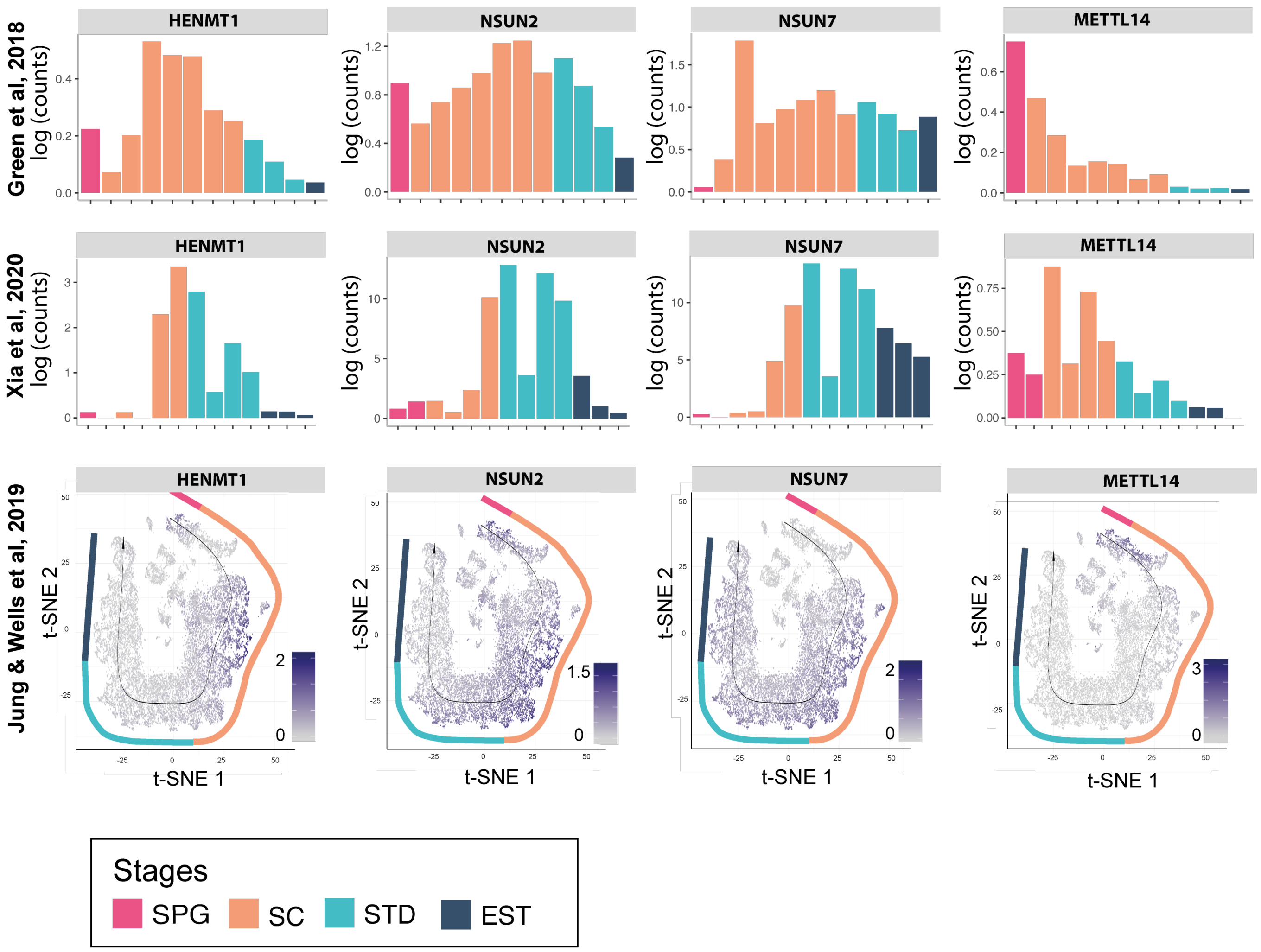

Fig. S10. Immunofluorescence of NSUN2 and NSUN7 RMPs in mouse testis. **(A)** Schematic of the area and orientation of the seminiferous tubules in the confocal images. **(B)** Localization of NSUN2 (red) and nucleolus marker Fibrillarin (green), with arrows indicating nucleoli and arrow heads the nucleoli of Sertoli cells. **(C)** Localization of NSUN7 (red) and chromatoid body marker DDX4 (green), with arrows indicating chromatoid body structures. **(D)** Localization of NSUN2 (red) and DDX4 (green). **(E)** IgG isotype controls. Nuclei were counter-stained with Hoechst 33342 (blue) and a merge of all channels is shown in the far-right column. Scale bar = 25 μm.

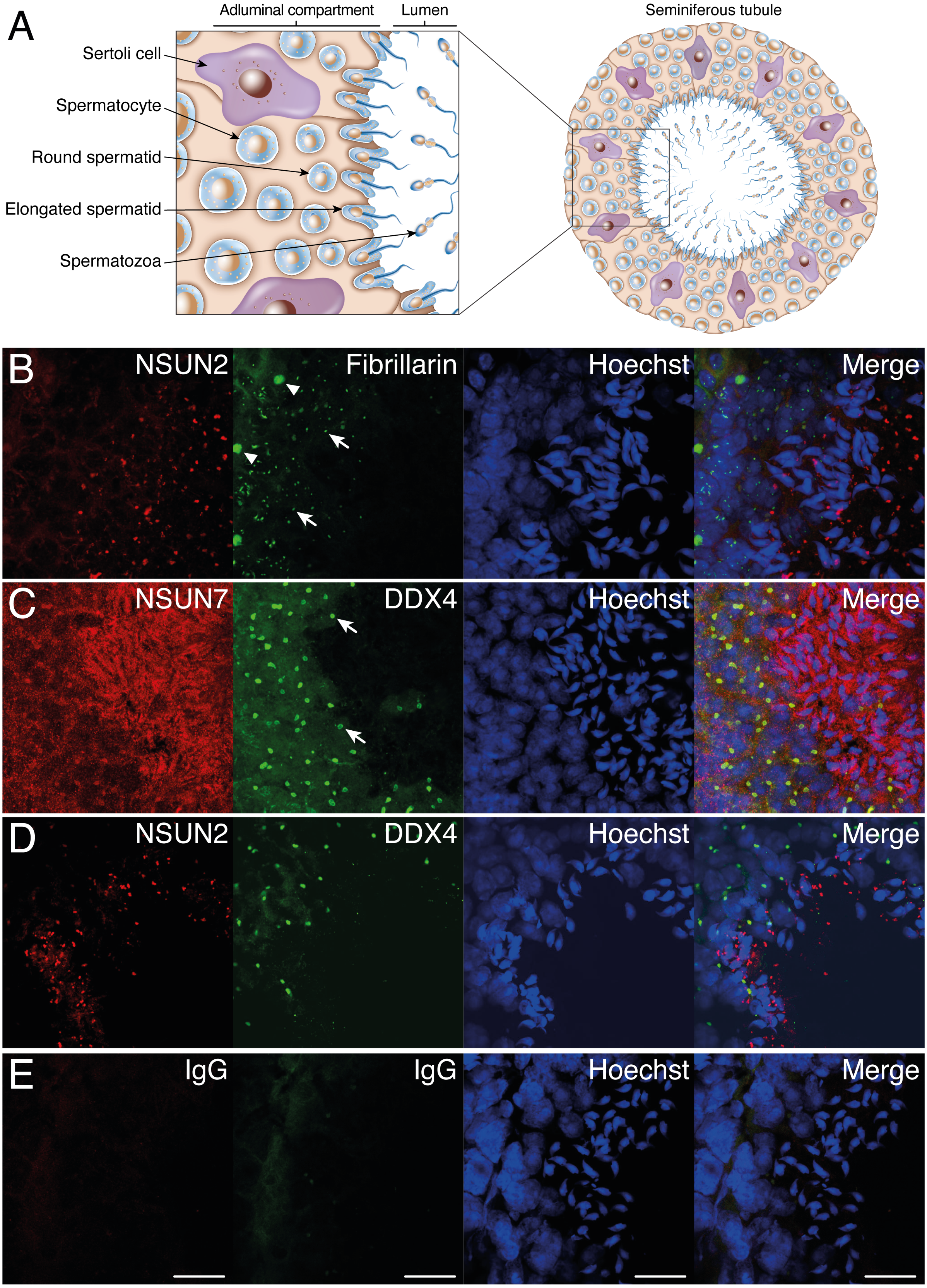

Fig. S11. Heatmap of the RMP expression changes between tumor and normal samples, across 28 cancer types. RMP expression is measured as log2 fold change (log2FC), using the mean differences of all patients. Positive (red) values indicate up-regulation in tumor, whereas negative (blue) values indicate down-regulation.

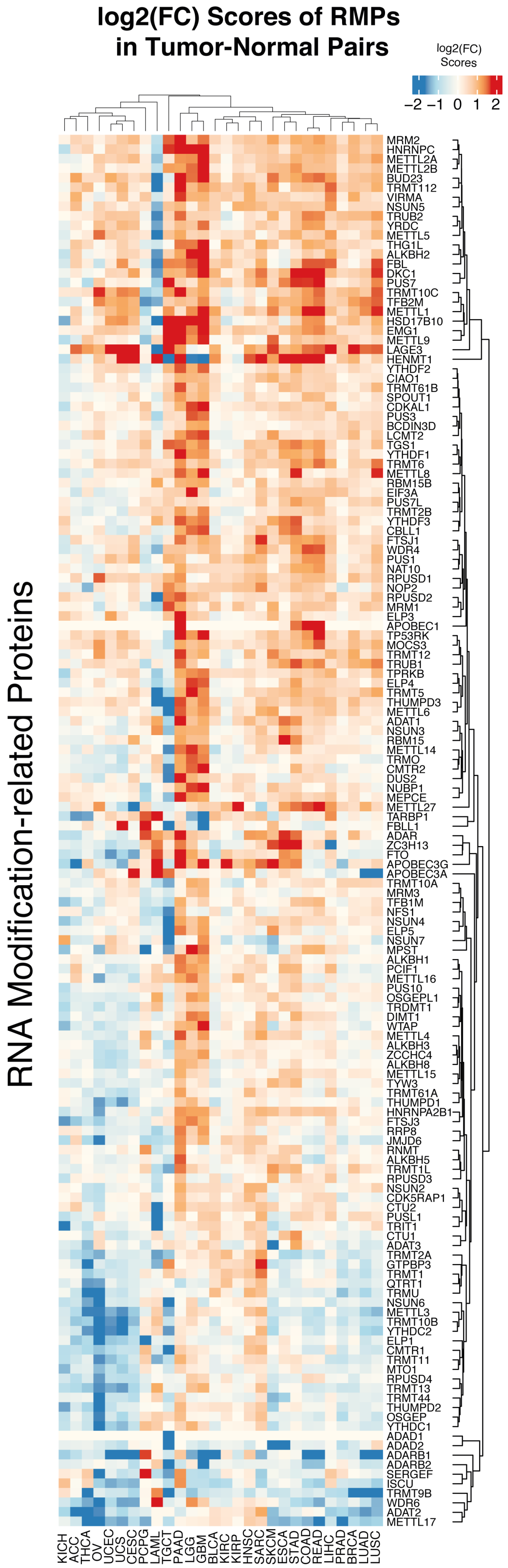

Fig. S12. Scatterplots showing expression levels of RMPs in matched tumor-normal samples for all 28 cancer types analyzed. Values represent median log(TPM) across all patients. RMPs are shown in black, unless they are significantly up-regulated (red) or down-regulated (blue). Non-RMP genes are shown in grey. Pearson correlation values are shown for each cancer type. See Table 1 for abbreviations used for each cancer type.

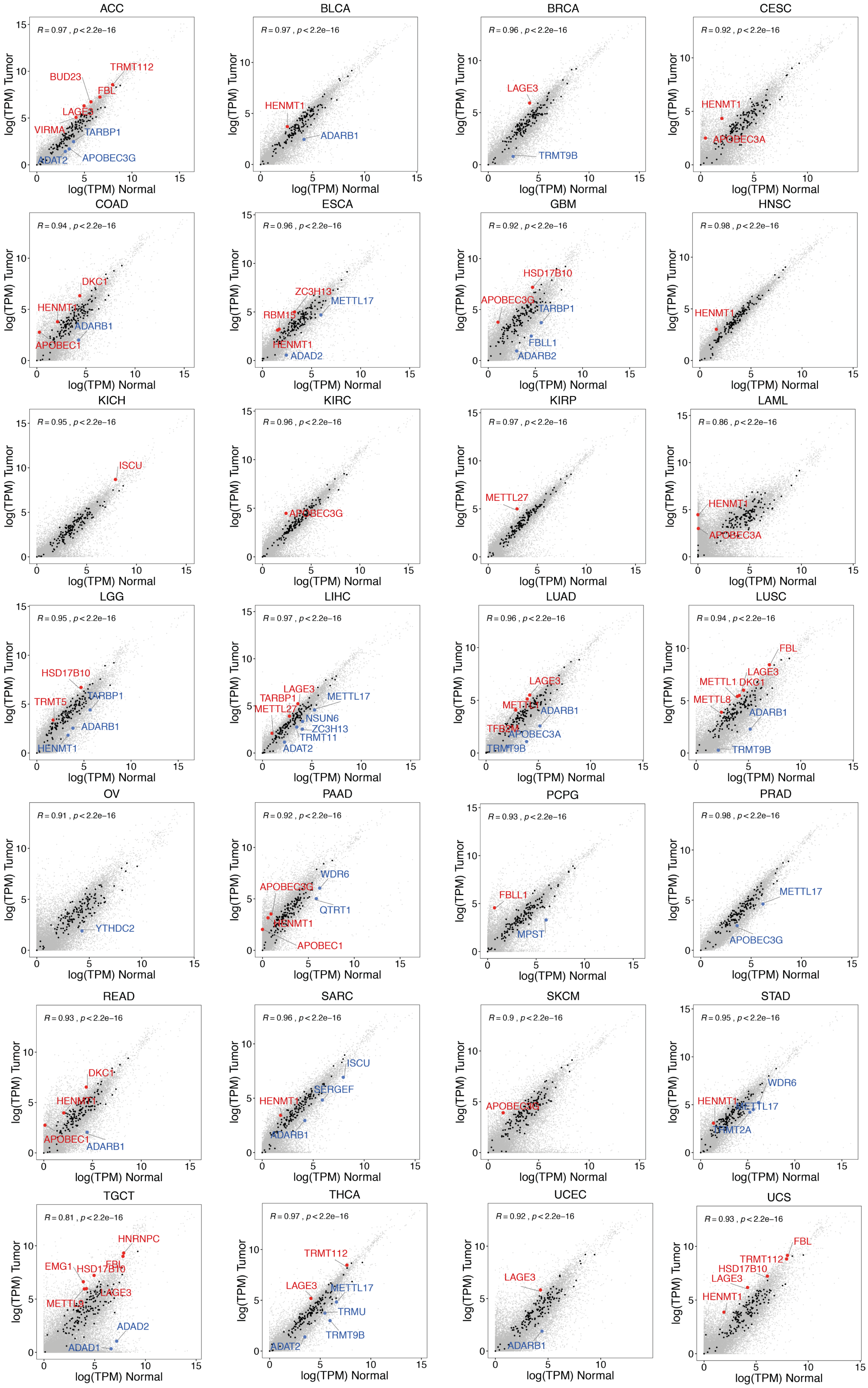

Fig. S13. (A) Analysis of LAGE3 (left) and HENMT1 (right) mRNA expression levels across different cancer types and stages, relative to normal tissue expression levels. Median log(TPM) values from each cancer stage were normalized to the median log(TPM) of the normal tissue. Dashed lines depict cancer types in which LAGE3 or HENMT1 is not dysregulated, whereas full lines are used for cancer types in which the gene is dysregulated. **(B)** Violin plots of mRNA expression levels of LAGE3 and HENMT1 in individual cancer types (COAD, BRCA and KIRC) and across stages. Each dot represents the mRNA expression levels of a different individual.

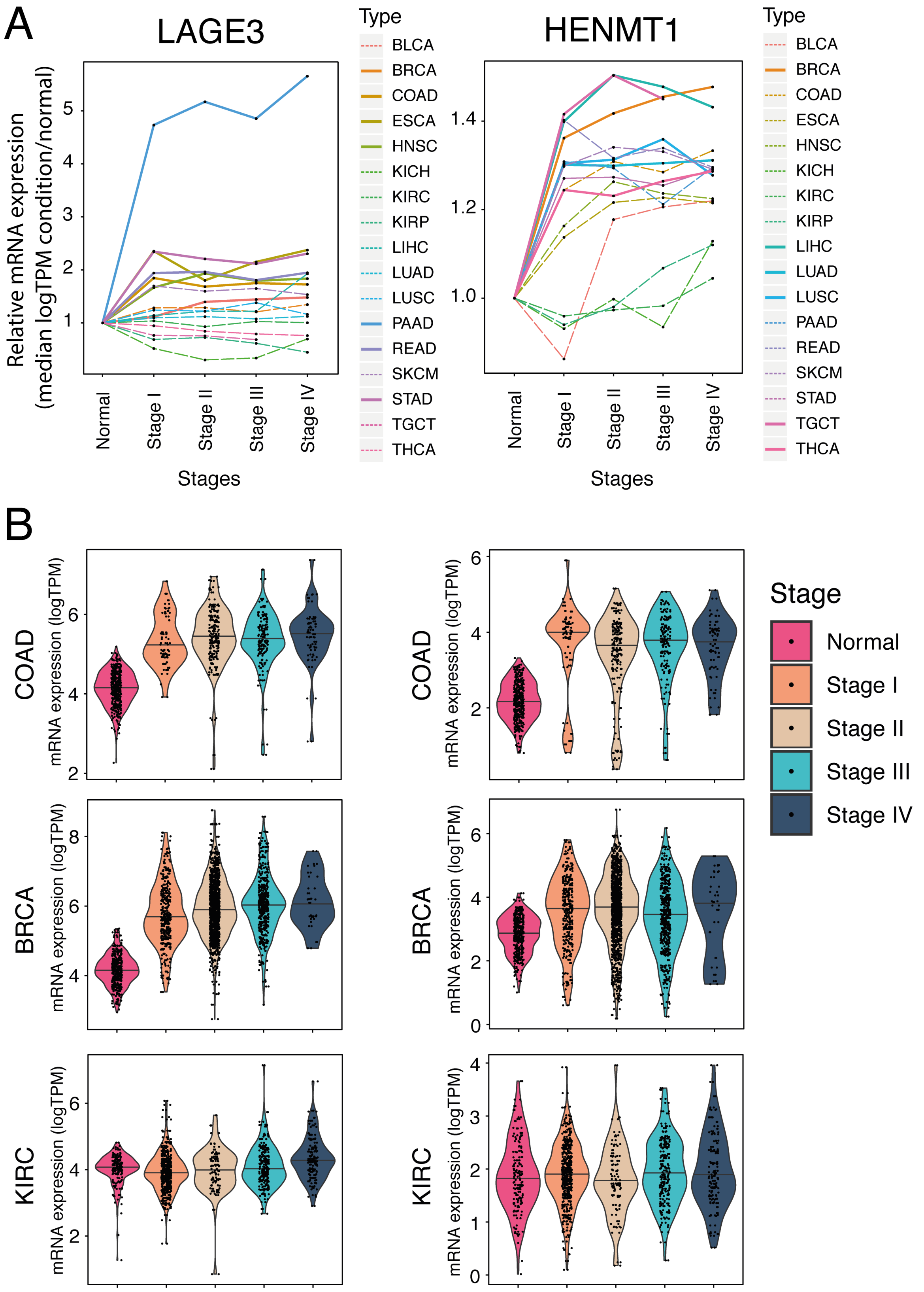

Fig. S14 . (A) Schematic representation of the layout of the Tissue Microarray (TMA) slide used for immunohistochemical analysis. **(B)** Overall staining pattern of TMAs using HENMT1 and LAGE3 antibodies. Brown color indicates specific staining of the antibody, whereas blue represents the hematoxylin counterstain. **(C)** TMA scores (mean of two independent blinded scorers) obtained for each cancer type included in the slide. Mean TMA score for each core is depicted with a horizontal line, whereas scores given to each individual core are shown as dots. P-values were computed using two-sided Wilcoxon tests.

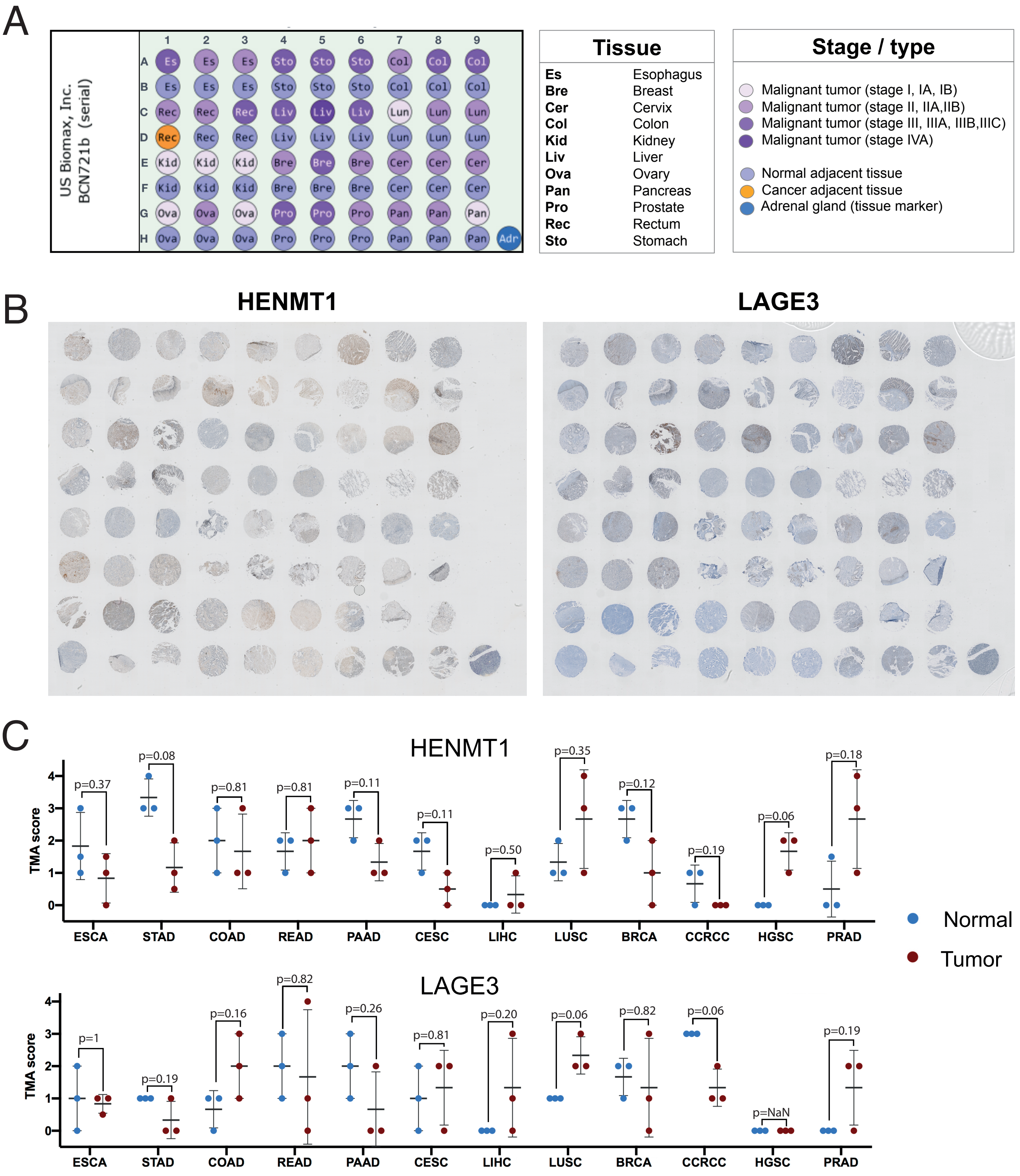

Fig. S14. Immunohistochemical staining of mouse testis and epididymis using isotype control rabbit IgG antibody (negative control). Brown color indicates antibody specific staining, whereas blue depicts hematoxylin counterstain.

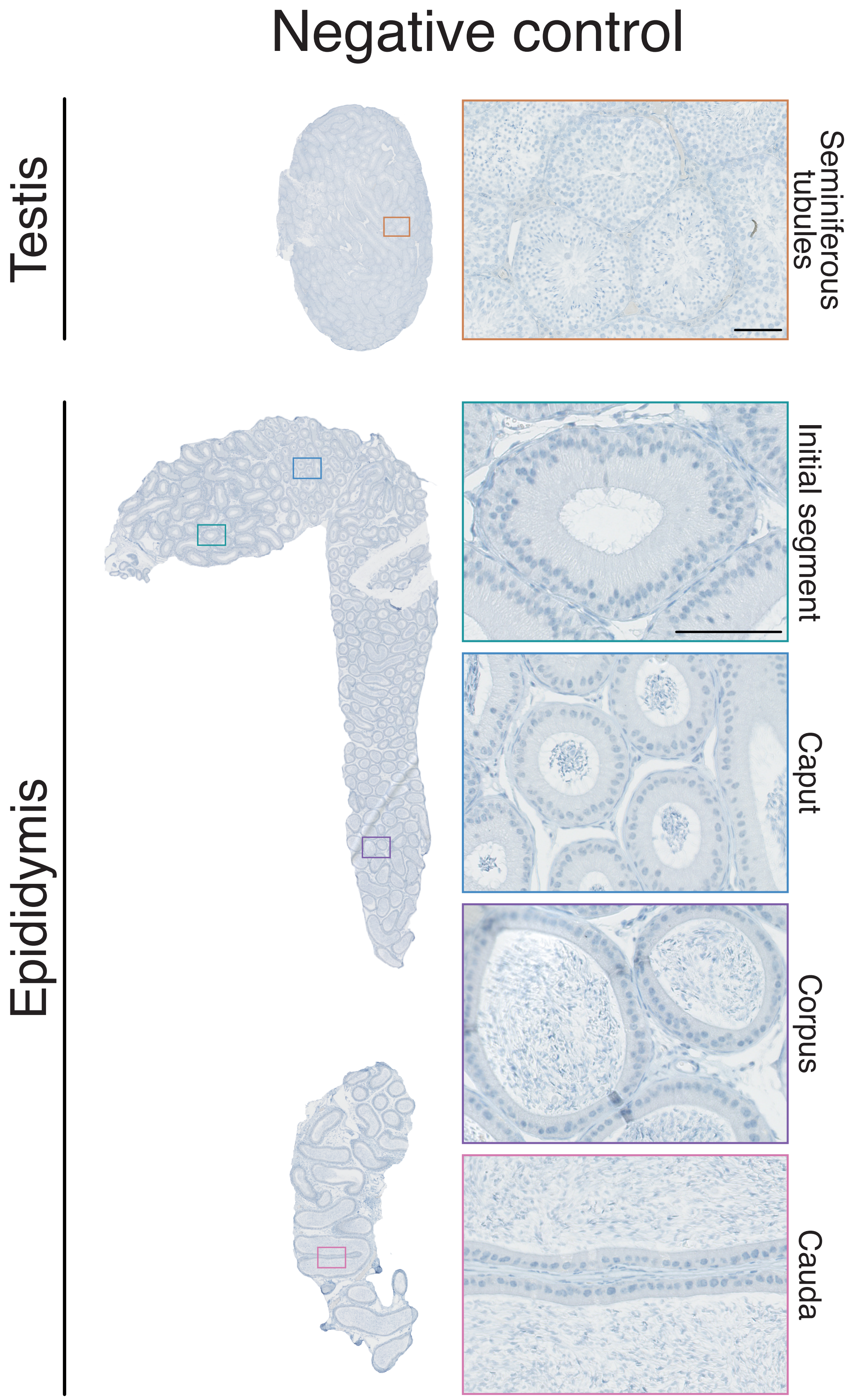

Supplement: Supplementary file 13 — Additional file 13: Figure S1. Expression analysis plots (Heatmap and PCA) of RMPs in Human and Mouse tissues. Figure S2. Quantitative real-time PCR of 8 RMPs expressed in four mouse tissues. Figure S3. Proteomics analysis of RMPs in human tissues. Figure S4. Expression of RMPs in Amniote and Primate species. Figure S5. Analysis of target specificity of tissue-specific and non-tissue-specific genes. Figure S6. Expression analysis of RMPs in mouse spermatogenesis and Immunohistochemical staining of HENMT1 in mouse testis and epididymis. Figure S7. Comparison of RMP expression changes during spermatogenesis using published single-cell RNA sequencing datasets (Green et al.,2018 and Xia et al., 2020). Figure S8. Comparison of RMP expression patterns during spermatogenesis, using the data published by Green et al., 2018 and Jung & Wells et al., 2019. Figure S9. Comparative analysis of mRNA expression levels of HENMT1, NSUN2, NSUN7 and METTL14 during spermatogenesis, extracted from 3 distinct single-cell RNAseq publicly available datasets. Figure S10. Immunofluorescence of NSUN2 and NSUN7 RMPs in mouse testis. Figure S11. Heatmap of the RMP expression changes (log2FC) between tumor and normal samples, across 28 cancer types. Figure S12. Scatterplots showing expression levels of RMPs in matched tumor-normal samples for all 28 cancer types analyzed. Figure S13. Tumor stage-specific RNA expression levels of LAGE3 and HENMT1. Figure S14. Immunohistochemical staining of Tissue microarray (TMA) with LAGE3 and HENMT1 antibodies. Figure S15. Immunohistochemical staining of mouse testis and epididymis using isotype control rabbit IgG antibody (negative control). [file 13059_2020_2009_MOESM13_ESM.pdf]
