# Supplementary material for: Integrative analyses of the RNA modification machinery reveal tissue- and cancer-specific signatures
Source: Genome Biol. 2020 May 7;21:97. doi: 10.1186/s13059-020-02009-z (PMC7204298; doi:10.1186/s13059-020-02009-z)
Supplement: Supplementary file 14 — Additional file 14. Review history. [file 13059_2020_2009_MOESM14_ESM.docx]

Review History

**First round of review**

**Reviewer 1**

**Are you able to assess all statistics in the manuscript, including the appropriateness of statistical tests used?**

No, I do not feel adequately qualified to assess the statistics.

**Comments to author:**

In their manuscript, Begik and colleagues perform comprehensive comparative analyses of all RNA modification enzymes known in humans across tissue, species and paired human normal and tumor samples. The authors find that many RNA modification enzymes underwent several duplication events during evolution resulting in a change of substrate-specificity and / or tissue specific expression. They then characterise testis-specific expression of some selected RMPs in greater detail and confirm cell type specific expression. Finally, they identify two RMPs, LAGE3 and HENMT1, to be dysregulated across all tumors analysed.

RNA modifications have emerged as important additional layer of regulation of most steps of gene expression and are therefore crucial regulators of cell fate and function. Although many of the over 170 known RNA modifications are known for decades, the corresponding enzymes mediating their formation have only more recently been identified. A full annotation of these enzymes is much-needed in the field and will significantly enhance our understanding of their functional roles in normal tissue development and diseases such as cancer. This is a highly important and timely study for the field. However, there are a couple of major concerns that need to be addressed.

Major comments:

1. Figure 2: The authors convincingly show that the analysed RMPs gained tissue-specific expression patterns with testis being the most distinctive tissue in terms of RMP expression. Spermatogenesis is quite unique in the sense that it contains stages of transcriptional arrest. Similarly, neural cells rely heavier than other tissues on post-transcriptional regulatory mechanisms of gene expression. How do the findings relate to the RNA targets and the functional roles of the RNAs in the tissues? In other words, do identified tissue-specific RMPs target specific classes of RNA?

2. The authors use single cell sequencing data to identify where in spermatogenesis the testis-specific RMPs are expressed. There are a couple of publications on mouse testis. How consistent is the cell stage-specific expression of the RMPs between the data? How does it compare to other species such as humans, flies …. ?

3. Page 8, line 44: "We found that NSUN7 is most highly expressed in chromatoid bodies of spermatocytes." To confirm the localization of NSUN2 at chromatoid bodies the authors need to provide co-staining with markers for chromatoid bodies. The same applies for the localization of NSUN2 in the nucleoli.

4. In Figure 4, the authors then measure the differential expression of the RMPs in over 10 000 tumor-normal paired samples and find that a couple of RMPs, including the less well-characterized HENMT1 and LAGE3, to be consistently dysregulated across all tumor types. While it is certainly interesting to find RMPs that are globally mis-regulated in all tumor types, the data are expected to be highly variable. The authors themselves demonstrate an often cell type- and tissue-specific expression of RMPs. Tumors also show high inter- and intra-heterogeneity and therefore, the conclusion that can be drawn from the analyses in Figure 4 seem quite limited. It might be more informative showing also some selected tumors on the single cell level, or at least stage the tumors, or stratify the tumor regarding treatment type and so on.

5. Some stainings of HENMT1 and LAGE3 in tumors are needed for the strong conclusions. In addition, it is unclear what this finding means for the tumor and the patients. Both RMPs (LAGE3 and HENMT1) seem to be both favourable and unfavourable prognostic markers depending on the tumor type (https://www.proteinatlas.org/).

Minor comments:

1. Page 8, line 16: Dnmt2 mediates 5-methylcytosine not methyladenosine.

**Reviewer 2**

**Are you able to assess all statistics in the manuscript, including the appropriateness of statistical tests used?**

No, I do not feel adequately qualified to assess the statistics.

**Comments to author:**

Are the methods appropriate to the aims of the study, are they well described, and are necessary controls included? Yes

Are the conclusions adequately supported by the data shown? No (the data showed great correlation, but not experimental validation at all. It would be great to experimentally test one or two examples identified)

Are sufficient details provided to allow replication and comparison with related analyses that may have been performed? Yes

Does the work represent a significant advance over previously published studies? Yes

Is the paper of broad interest to others in the field, or of outstanding interest to a broad audience of biologists? yes

"In Begik et al., the authors compiled an array of previously-generated sequencing data related to the expression levels several RNA modification-related proteins (RMPs). Using these data, the authors perform a phylogenetic analysis of these RMPs and correlate the evolutionary timing of gene duplication events with a change in substrate specificity of these proteins. In addition, the authors find a striking expression phenotype of several RMPs in the testes, and find a further correlation between RMPs and different stages of spermatogenesis. These findings are then validated with immunohistochemistry. Finally, the authors use their data to evaluate their annotated RMPs as potential cancer biomarkers, and find several whose expression is correlated with survival in two types of cancers. Of these, specific focus is given to HENMT1 and LAGE3.

While these findings are certainly compelling and can provide a framework for future studies, the reviewer provides the following comments:

*	Page 5- line 4, "post-transcriptional" is not the most appropriate, many of the modifications are shown co-transcriptional installed.

*	Page 6 - The authors should give some speculation as to why these gene duplication events are correlated with a change in function

*	Page 6 line 55 - The authors do not address any functional implications of gene expression patterns following this point, and thus it's reference at this point of the study is confusing

*	Page 8 - The authors use of IHC is an excellent example of backing up in silico findings with in vivo validation

*	Page 9 - The authors cite an example of a FTO inhibitor being used as a cancer treatment, but later in the manuscript they point out that their study contradicts the efficacy of such inhibitors. The authors should address this contradiction

*	The data as presented to this reviewer, are blurry and hard to read. I understand this may be a file compression issue, but the authors should take care that their figures are high resolution

*	Overall, figures in my download version were very unclear, the resolution need to be enhanced by A LOT

**Authors Response**

**Point-by-point responses to the reviewers’ comments:**

Reviewer #1:

In their manuscript, Begik and colleagues perform comprehensive comparative analyses of all RNA modification enzymes known in humans across tissue, species and paired human normal and tumor samples. The authors find that many RNA modification enzymes underwent several duplication events during evolution resulting in a change of substrate-specificity and / or tissue specific expression. They then characterise testis-specific expression of some selected RMPs in greater detail and confirm cell type specific expression. Finally, they identify two RMPs, LAGE3 and HENMT1, to be dysregulated across all tumors analysed.

RNA modifications have emerged as an important additional layer of regulation of most steps of gene expression and are therefore crucial regulators of cell fate and function. Although many of the over 170 known RNA modifications are known for decades, the corresponding enzymes mediating their formation have only more recently been identified. A full annotation of these enzymes is much-needed in the field and will significantly enhance our understanding of their functional roles in normal tissue development and diseases such as cancer. This is a highly important and timely study for the field. However, there are a couple of major concerns that need to be addressed.

Major comments:

1. Figure 2: The authors convincingly show that the analysed RMPs gained tissue-specific expression patterns with testis being the most distinctive tissue in terms of RMP expression. Spermatogenesis is quite unique in the sense that it contains stages of transcriptional arrest. Similarly, neural cells rely heavier than other tissues on post-transcriptional regulatory mechanisms of gene expression. How do the findings relate to the RNA targets and the functional roles of the RNAs in the tissues? In other words, do identified tissue-specific RMPs target specific classes of RNA?

*To address the reviewer’s question, we first examined whether tissue-specific RMPs target specific classes of RNA, compared to non-tissue-specific RMPs. We find that non-tissue specific RMPs mainly target tRNAs, rRNAs and mRNAs. Tissue-specific RMPs also target these RNAs, they also target a higher proportion of small non-coding RNAs, including piRNA and eRNA (Figure S5A).*

*We then extended this analysis to address the second question of the reviewer, i.e. whether tissue-specific RMPs of individual tissues also show distinct RNA targets. From the set of 32 tissues analyzed, only tissues with at least 5 tissue-specific RMPs with known RNA targets were kept for further analysis. The final list of tissues included testis (n=20), liver (n=6), bone marrow (n=6) and cerebellum (n=8), which were used to examine whether the tissue-specific RMPs of distinct tissues had distinct RNA targets. We found that liver and bone marrow mainly target tRNAs, rRNAs and mRNAs. In contrast, cerebellum and testis displayed a larger proportion of tissue-specific RMPs that target distinct families of small non-coding RNAs, in agreement with the reviewer’s comment that both testis and neural tissues rely more than other tissues in post-transcriptional regulatory mechanisms. These results have now been included in the manuscript (Figure S5B).*

2. The authors use single cell sequencing data to identify where in spermatogenesis the testis-specific RMPs are expressed. There are a couple of publications on mouse testis. How consistent is the cell stage-specific expression of the RMPs between the data? How does it compare to other species such as humans, flies …. ?

*Following the reviewer’s suggestion, we have performed the analysis on two additional mouse spermatogenesis RNAseq datasets (Jung & Wells et al., eLife 2019 and Xia et al, Cell 2020). We find that all three datasets (the original one used, Green et al., 2018 and the two mentioned above) show high similarities in terms of RMP expression profiles. Specifically, we find that the expression patterns of RMPs identified in each expression group (Group 1,2,3,4) are highly consistent with the results obtained using the first study that was used in our work, including those RMPs whose expression was validated using IHC. Results have been added in the form of Supplementary Figure S7 (analysis of Xia et al., 2020) and Figure S8 (analysis of Jung & Wells et al, 2019).*

3. Page 8, line 44: "We found that NSUN7 is most highly expressed in chromatoid bodies of spermatocytes." To confirm the localization of NSUN2 at chromatoid bodies the authors need to provide co-staining with markers for chromatoid bodies. The same applies for the localization of NSUN2 in the nucleoli.

*Following the reviewer’s suggestion, we have now performed immunofluorescence (IF) assays to confirm subcellular localization of NSUN7 and NSUN2. Specifically, we have performed IF with: (i) anti-fibrillarin (FBL), which is commonly used in literature as nucleolar marker and (ii) anti-DDX4, which is reported in literature as a marker for chromatoid bodies (Kotaja et al. 2006). Our results were unable to confirm co-localization between NSUN2 with DDX4 (chromatoid bodies) or with FBL (nucleoli). Similarly, NSUN7 did not co-localize DDX4. We have now added these results in Supplementary Figure S9 and have also amended and discussed these results in the main text, where we are now not suggesting a specific subcellular localization as we were unable to confirm using immunofluorescence. We thank the reviewer for his comment and suggestion, as it has helped us to improve the accuracy of our work.*

4. In Figure 4, the authors then measure the differential expression of the RMPs in over 10 000 tumor-normal paired samples and find that a couple of RMPs, including the less well-characterized HENMT1 and LAGE3, to be consistently dysregulated across all tumor types. While it is certainly interesting to find RMPs that are globally mis-regulated in all tumor types, the data are expected to be highly variable. The authors themselves demonstrate an often cell type- and tissue-specific expression of RMPs. Tumors also show high inter- and intra-heterogeneity and therefore, the conclusion that can be drawn from the analyses in Figure 4 seem quite limited. It might be more informative showing also some selected tumors on the single cell level, or at least stage the tumors, or stratify the tumor regarding treatment type and so on.

*Following the reviewer’s suggestion, we have now reanalyzed the results using stage-specific expression profiles for LAGE3 and HENMT1. We find that overall expression patterns in the tumors with stage information are increased with respect to normal tissues, in all stages analyzed, i.e. that the dysregulation occurs in all stages, compared to the normal tissue. We have now included these results as Supplementary Figure S12.*

5. Some stainings of HENMT1 and LAGE3 in tumors are needed for the strong conclusions. In addition, it is unclear what this finding means for the tumor and the patients. Both RMPs (LAGE3 and HENMT1) seem to be both favourable and unfavourable prognostic markers depending on the tumor type (https://www.proteinatlas.org/).

*Following the reviewer’s suggestion, we have now performed immunohistochemistry assays on tissue microarrays (TMAs) of matched tumor and normal samples from multiple human tissues, using both anti-LAGE3 and anti-HENMT1 antibodies. We find that both HENMT1 and LAGE3 are upregulated in specific tissue types, although the p-value of the up-regulation is not statistically significant, partly due to the low number of cores per cancer type (n=3) as well as to the variability of analyzing differently graded cancer types (Figure 5A and Figure S13). Nevertheless, we find that expression of both of them appears to be dysregulated in multiple cancer types, in agreement with our observations using transcriptomic analyses.*

*The reviewer’s also mentions that both LAGE3 and HENMT1 seem to be both favourable and unfavourable prognostic markers, depending on the cancer type. We would like to note that while this is true, this does not exclude its value as prognostic marker for a specific tumor type.*

Minor comments:

1. Page 8, line 16: Dnmt2 mediates 5-methylcytosine not methyladenosine.

*We have now corrected the typo in the manuscript. We thank the reviewer for pointing us to it.*

Reviewer #2:

Are the methods appropriate to the aims of the study, are they well described, and are necessary controls included? Yes

Are the conclusions adequately supported by the data shown? No (the data showed great correlation, but not experimental validation at all. It would be great to experimentally test one or two examples identified)

Are sufficient details provided to allow replication and comparison with related analyses that may have been performed? Yes

Does the work represent a significant advance over previously published studies? Yes

Is the paper of broad interest to others in the field, or of outstanding interest to a broad audience of biologists? Yes

*The reviewer suggests that additional experimental validation should be included to further support the conclusions of the work. Specifically, we have now included the following additional experiments:*

*1) For tissue-specific expression patterns: Firstly, we have performed both qRT-PCR of tissue-specific/enriched and non-tissue enriched RMPs. Our results validate the restricted tissue specific RMP expression patterns, and are in agreement with the RNAseq data. These results have been added in Figure S2. Secondly, we have analyzed publicly available proteomics data in multiple human tissues (Kim et al., Nature 2014). We find that testis is the most distinctive tissue in terms of RMP expression patterns, also at the protein level (Figure S3)*

*2) For spermatogenesis data analysis: Firstly, we have analyzed two additional published mouse spermatogenesis scRNAseq datasets (Xia et al., Cell 2020; Jung et al. eLife 2019), finding that the results are consistent in across all 3 datasets (Figure S7 and S8). Secondly, we have performed immunofluorescence assays to further dissect the subcellular localization of specific RMPs (NSUN2 and NSUN7) in testis tissues. We examined whether these two proteins might be present in either chromatoid bodies or nucleolus using well-characterized markers (DDX4 and FBL) (Figure S9). However, we did not find clear co-localization between NSUN2 or NSUN7 with neither of the two markers.*

*3) For tumor-normal data analysis: Firstly, we have reanalyzed the RNAseq TCGA data considering separately differently graded tumors, showing that the upregulation of LAGE3 and HENMT1 in specific cancer types is relatively consistent across stages (Figure S12), although it tends to increase with later stages. Secondly, we have performed immunohistochemistry assays on paraffin-embedded patient-derived samples (Tissue Microarrays) across a range of cancer types and diverse tumor grades, including normal tissue samples as control in the same slide. TMAs were stained both anti-LAGE3 and anti-HENMT1. Our results show that both LAGE3 and HENMT1 are up-regulated in specific tumor types (Figure 5A). Unfortunately, the variability among cancer grades across the tumor cores, together with the low number of cores per cancer type (n=3) did not allow to obtain significant p-values (Figure S13). Nevertheless, our results suggest that LAGE3 and HENMT1 have altered expression levels in specific cancer types, in agreement with the RNAseq data.*

* Page 5- line 4, "post-transcriptional" is not the most appropriate, many of the modifications are shown co-transcriptional installed.

*We have removed the term post-transcriptional in this sentence. We thank the reviewer for pointing us to it.*

* Page 6 - The authors should give some speculation as to why these gene duplication events are correlated with a change in function

*We have now added a comment on changes in gene function in the manuscript. We thank the reviewer for the suggestion.*

* Page 6 line 55 - The authors do not address any functional implications of gene expression patterns following this point, and thus it's reference at this point of the study is confusing

*We understand that this sentence could lead to confusion, as it was mentioned without context or additional discussion. We have now amended the sentence and removed the mention to ‘possible functional implications’.*

* Page 8 - The authors use of IHC is an excellent example of backing up in silico findings with in vivo validation

*We thank the reviewer for his/her comment.*

* Page 9 - The authors cite an example of a FTO inhibitor being used as a cancer treatment, but later in the manuscript they point out that their study contradicts the efficacy of such inhibitors. The authors should address this contradiction

*The reviewer makes an interesting observation of an apparent contradiction: FTO inhibitors are actively being investigated to treat LAML; however, in our study we do not find FTO to be dysregulated in LAML.*

*While at first glance this may seem contradictory, our results do not contradict the efficacy of such inhibitors. Rather, our results show that FTO is not significantly upregulated in LAML once the variance across tumor-normal data is taken into account. However, that does not exclude the fact that FTO treatment may be a successful anti-tumoral therapy.*

* The data as presented to this reviewer, are blurry and hard to read. I understand this may be a file compression issue, but the authors should take care that their figures are high resolution

*We are unsure about which may have been the problem with the figure visualization, as we have uploaded all figures with high resolution. We believe that this might be related to the final PDF that is automatically generated by the system.*

*We have now uploaded all main figures in high resolution individually, both in PNG and PDF. We would be happy to provide them in any other form that may be needed.*

* Overall, figures in my download version were very unclear, the resolution need to be enhanced by A LOT

*Please see previous comment.*

**Second round of review**

**Reviewer 1**

The authors have addressed my original concerns by providing more analyses or clarifying the text. The generation of a detailed list of annotated RMPs is an important contribution to the field. The authors use their findings to (i) demonstrate heterogeneous expression of the RMPs within a tissue using the mouse testis as an example, and (ii) identify RMPs mis-regulated in cancer. The conclusions from these analyses remain largely descriptive and should be toned down (see specific comments). In particular, the statement that METTL3 and FTO are not differently expressed in most cancer types once a properly scaling is performed is quite provocative given that at least some of studies in question have performed detailed cell type-specific and functional analyses. In addition, the claim in the abstract that in contrast, LAGE3 and HENMT1 are dys-regulated in many cancers is misleading as the selection criteria was only a minimum 2 out 28 different cancers. Because the study tackles two very different, yet highly complex aspects, it remains largely superficial. Therefore, I strongly recommend removing some of the very strong conclusions.

Specific comments:

1. In the authors’ response to my question how consistent the cell stage-specific expression of the RMPs is compared to other published datasets, the authors provided the same analyses using publicly available datasets from mouse. They conclude “similar expression patterns were observed when analyzing other publicly available single cell mouse spermatogenesis RNAseq datasets [60,61] (Figure S7 and S8). However, in the provided figures Group 3 and Group 4 seem quite substantially different. Since the chosen examples of NSUN7, NSUN2 and HENMT1 all belong to these groups, the authors should show at least their expression in the different datasets.

2. In the original version the authors claimed on Page 8, line 44: “We found that NSUN7 is most highly expressed in chromatoid bodies of spermatocytes.” On the same page line 51 they claimed: “On the other hand, NSUN2 displayed high expression levels in the nucleolus of the spermatogonial cells and spermatocytes”. In response to my comment to confirm this subcellular localization, the authors now provide IF staining including DDX4 and Fibrillarin to label the chromatoid body and nucleolus respectively. They conclude “ …We found that NSUN2 was expressed in the lumen as well as the adluminal compartment. Surprisingly, we found that the expression of NSUN2 and DDX4 was quasi mutually exclusive, being DDX4 expressed in earlier stages of spermatogenesis, and NSUN2 being expressed in later stages. We did not observe colocalization of NSUN2 with DDX4, in contrast to previous reports [64].” The staining in Figure S9 is not convincing and seems to identify NSUN2 mainly in the spermatozoa. Furthermore, NSUN2 did not co-localize with Fibrillarin. These data undermine the results shown in Figure 3D. I recommend omitting all data and statements regarding subcellular localizations.

3. The authors further claim “ METTL14 was also found to be highly expressed in early spermatogenesis and down-regulated during the subsequent stages (Figure 3D, right panels)”. This statement is incorrect as Figure 3D, right panels show in general less cells, yet a very strong staining. The author should reword this section.

4. Along the same line, the statement “Finally, HENMT1 was mainly expressed in round and elongating spermatocytes, where it is accumulated in the chromatoid bodies (Figure S6E, see also Figure S9)”, is also not accurately describing the results. No such conclusion can be drawn from the figures provided as no co-localization has been shown, and Figure S6E hardly shows any staining at all. While I do appreciate that the authors try to validate their findings with tissue stainings, the conclusions are too strong and the whole section needs to be toned down.

5. The authors now provide confirming stainings of HENMT1 and LAGE3 in tumors. In the majority of cases, it seems not a general but quite cell type-specific regulation. This seem to confirm my previous comment that tumors also show high inter- and intra-heterogeneity for the RMP expression and therefore, the conclusion that can be drawn from the analyses are still limited.

**Reviewer 2**

The authors have addressed my questions.

**Authors Response**

**Point-by-point responses to the reviewers’ comments:**

Reviewer #1:

The authors have addressed my original concerns by providing more analyses or clarifying the text. The generation of a detailed list of annotated RMPs is an important contribution to the field. The authors use their findings to (i) demonstrate heterogeneous expression of the RMPs within a tissue using the mouse testis as an example, and (ii) identify RMPs mis-regulated in cancer. The conclusions from these analyses remain largely descriptive and should be toned down (see specific comments). In particular, the statement that METTL3 and FTO are not differently expressed in most cancer types once a properly scaling is performed is quite provocative given that at least some of studies in question have performed detailed cell type-specific and functional analyses. In addition, the claim in the abstract that in contrast, LAGE3 and HENMT1 are dys-regulated in many cancers is misleading as the selection criteria was only a minimum 2 out 28 different cancers. Because the study tackles two very different, yet highly complex aspects, it remains largely superficial. Therefore, I strongly recommend removing some of the very strong conclusions.

*We thank the reviewer for his/her time and his/her comments.*

*The reviewer mentions that the claim in the abstract stating that “LAGE3 and HENMT1 are dysregulated in many cancers” is misleading, as the selection criteria used was a an RMP had to be dysregulated in a minimum of 2 out of 28 cancers (Figure 4C). While the reviewer is correct with regards to the selection criteria, HENMT1 and LAGE3 appear in the top of the ranked list of “top-dysregulated RMPs” using this criteria. Specifically, we find that HENMT1 is up-regulated in 11/28 cancer types studies, and LAGE3 is dysregulated in 9/28 cancer types studied (Figure 4D).*

*However, we do agree that some of our conclusions could be toned down, and we have now amended some specific sentences throughout the manuscript accordingly (please see individual responses below to the specific comments).*

Specific comments:

1. In the authors’ response to my question how consistent the cell stage-specific expression of the RMPs is compared to other published datasets, the authors provided the same analyses using publicly available datasets from mouse. They conclude “similar expression patterns were observed when analyzing other publicly available single cell mouse spermatogenesis RNAseq datasets [60,61] (Figure S7 and S8). However, in the provided figures Group 3 and Group 4 seem quite substantially different. Since the chosen examples of NSUN7, NSUN2 and HENMT1 all belong to these groups, the authors should show at least their expression in the different datasets.

*Following the reviewer’s suggestion, we have now included the expression levels across spermatogenesis stages for NSUN7, NSUN2, HENMT1 and METTL14, for the 3 datasets used in this work (Xia et al., 2020; Green et al. 2018, Jung & Wells 2019). These results have now been added in the form of supplementary Figure S9.*

2. In the original version the authors claimed on Page 8, line 44: “We found that NSUN7 is most highly expressed in chromatoid bodies of spermatocytes.” On the same page line 51 they claimed: “On the other hand, NSUN2 displayed high expression levels in the nucleolus of the spermatogonial cells and spermatocytes”. In response to my comment to confirm this subcellular localization, the authors now provide IF staining including DDX4 and Fibrillarin to label the chromatoid body and nucleolus respectively. They conclude “ …We found that NSUN2 was expressed in the lumen as well as the adluminal compartment. Surprisingly, we found that the expression of NSUN2 and DDX4 was quasi mutually exclusive, being DDX4 expressed in earlier stages of spermatogenesis, and NSUN2 being expressed in later stages. We did not observe colocalization of NSUN2 with DDX4, in contrast to previous reports [64].” The staining in Figure S9 is not convincing and seems to identify NSUN2 mainly in the spermatozoa. Furthermore, NSUN2 did not co-localize with Fibrillarin. These data undermine the results shown in Figure 3D. I recommend omitting all data and statements regarding subcellular localizations.

*Following the reviewer’s suggestion, in our previous reviewed version of our manuscript we included immunofluorescence assays, to better address the subcellular localisation of NSUN2 and NSUN7 proteins. From these results, we found that NSUN2 and DDX4 were not co-localizing, and therefore we have excluded subcellular localization statements in our IHC results, and have only discussed subcellular localisation based on the results obtained using immunofluorescence assays.*

*However, we don’t agree with the reviewer’s comment about Figure S9 results undermining those in Figure 3D. In our opinion, we do find that IHC and IF stainings of NSUN7 and NSUN2 in seminiferous tubules are relatively consistent (see image in the attached word document named "Answers to Reviewers".This image has been built by zooming in the IHC images from Figure 4D, whereas the NSUN2 IF images have been extracted from Figure S9, and the NSUN7 IF image has been extracted from the raw IF data that we have deposited in FigShare. We can include this figure in the manuscript if the reviewer and/or editor considers it best for clarification of the results.*

*We have also removed the “merge” line from Figure S9 as we believe this might have caused confusion of which cells corresponded to sperm and which corresponded to spermatids.*

3. The authors further claim “ METTL14 was also found to be highly expressed in early spermatogenesis and down-regulated during the subsequent stages (Figure 3D, right panels)”. This statement is incorrect as Figure 3D, right panels show in general less cells, yet a very strong staining. The author should reword this section.

*We thank the reviewer for their comment and have now added a sentence to the manuscript to clarify the METTL14 IHC staining results, which we maintain supports the RNA sequencing data. We see METTL14-positive cells in the seminiferous tubules, but not in the very interior where round spermatids and elongated spermatids are located (black arrow heads). We also find that spermatozoa, which are located in the lumen of the seminiferous tubules and epididymis (red arrow heads), are unstained. The strongly stained cells found in the epithelium of the epididymis (blue arrows) are somatic cells (tall columnar cells and shorter basal cells).*

4. Along the same line, the statement “Finally, HENMT1 was mainly expressed in round and elongating spermatocytes, where it is accumulated in the chromatoid bodies (Figure S6E, see also Figure S9)”, is also not accurately describing the results. No such conclusion can be drawn from the figures provided as no colocalization has been shown, and Figure S6E hardly shows any staining at all. While I do appreciate that the authors try to validate their findings with tissue stainings, the conclusions are too strong and the whole section needs to be toned down.

*We agree with the above comment and removed the mention of chromatoid body localization of HENMT1.*

5. The authors now provide confirming stainings of HENMT1 and LAGE3 in tumors. In the majority of cases, it seems not a general but quite cell type-specific regulation. This seems to confirm my previous comment that tumors also show high inter- and intra-heterogeneity for the RMP expression and therefore, the conclusion that can be drawn from the analyses are still limited.

*When designing our IHC experiments of human tumor-normal matched samples, we decided to choose a slide that would contain a wide variety of tumor types. However, the direct consequence of this choice is that there are only 3 tumor cores per tissue type, leading to decreased statistical power. However, we considered that exploring a wide variety of tumor-normal types was more relevant than getting significant p-values for few selected cancer types. Therefore, we consider that the fact that few tumors show statistically significant differences is partly due to intra- and inter-heterogeneity of the RMP expression, as the reviewer suggests, but also due to the low number of cores per tissue that we were able to include in our analyses. We hope that the results from our work will be followed by future work re-examining this question (i.e. focus on fewer tumor types, but with large number of cores per tumor type), thus being able to dissect the cell-type specificity in the regulation of RMPs within tissues.*

Reviewer #2:

The authors have addressed my questions.

*We thank the reviewer again for his/her time in reviewing our work.*
